# Chromatin accessibility is associated with CRISPR-Cas9 efficiency in the zebrafish (*Danio rerio*)

**DOI:** 10.1371/journal.pone.0196238

**Published:** 2018-04-23

**Authors:** Meri I. E. Uusi-Mäkelä, Harlan R. Barker, Carina A. Bäuerlein, Tomi Häkkinen, Matti Nykter, Mika Rämet

**Affiliations:** 1 Laboratory of Experimental Immunology, BioMediTech Institute and Faculty of Medicine and Life Sciences, University of Tampere, Tampere, Finland; 2 Laboratory of Anatomy, Faculty of Medicine and Life Sciences, University of Tampere, Tampere, Finland; 3 Laboratory of Computational Biology, BioMediTech Institute and Faculty of Medicine and Life Sciences, University of Tampere, Tampere, Finland; 4 PEDEGO Research Unit, Medical Research Center Oulu, and Department of Children and Adolescents, Oulu University Hospital, Oulu, Finland; 5 Department of Pediatrics, Tampere University Hospital, Tampere, Finland; Texas A&M University, UNITED STATES

## Abstract

CRISPR-Cas9 technology is routinely applied for targeted mutagenesis in model organisms and cell lines. Recent studies indicate that the prokaryotic CRISPR-Cas9 system is affected by eukaryotic chromatin structures. Here, we show that the likelihood of successful mutagenesis correlates with transcript levels during early development in zebrafish (*Danio rerio*) embryos. In an experimental setting, we found that guide RNAs differ in their onset of mutagenesis activity *in vivo*. Furthermore, some guide RNAs with high *in vitro* activity possessed poor mutagenesis activity *in vivo*, suggesting the presence of factors that limit the mutagenesis *in vivo*. Using open access datasets generated from early developmental stages of the zebrafish, and guide RNAs selected from the CRISPRz database, we provide further evidence for an association between gene expression during early development and the success of CRISPR-Cas9 mutagenesis in zebrafish embryos. In order to further inspect the effect of chromatin on CRISPR-Cas9 mutagenesis, we analysed the relationship of selected chromatin features on CRISPR-Cas9 mutagenesis efficiency using publicly available data from zebrafish embryos. We found a correlation between chromatin openness and the efficiency of CRISPR-Cas9 mutagenesis. These results indicate that CRISPR-Cas9 mutagenesis is influenced by chromatin accessibility in zebrafish embryos.

## Introduction

Since its discovery in *Streptococcus pyogenes*, the CRISPR-Cas9 (Clustered regularly interspaced short palindromic repeats–CRISPR associated 9) system has been extensively applied to modify the eukaryotic genome in a targeted manner [1,2]. CRISPR-Cas9 technology takes advantage of the bacterial Cas9 endonuclease, which generates a double stranded break in its DNA target [1]. The repair of the break by the error prone repair machinery of non-homologous end joining often leads to the incorporation of mutations and permanent modifications to the genome [2].

Cas9 is directed to bind its target sequence by a single chimeric guide RNA molecule (sgRNA), which recognizes an approximately 20 nucleotide target site, followed by the three nucleotide protospacer adjacent motif (PAM)-sequence (5’-NGG-3’) [1–3]. The sgRNA sequence is considered the limiting step in mutagenesis design, as the genomic target site needs to be unique. An optimal GC-content and specific nucleotides at key positions in the target sequence can also alter the efficiency and the specificity of mutagenesis [4–8]. The efficiency and unspecific, off-target binding of the nuclease are not easy to predict. As a result, multiple algorithms and online tools have been created for the identification of guide RNA targets with optimal Cas9 loading scores and the least amount of off-targets [9–17]. However, the *in silico* predictions do not always correlate with the observed mutagenesis efficiency and specificity [11,18–20].

Eukaryotic gene expression is regulated at the epigenetic level by packing of DNA into nucleosomes, which are formed by wrapping 146bp of DNA around a histone octamer [21]. These eukaryotic chromatin structures fundamentally differ from bacterial DNA packing, and being a prokaryotic enzyme, it is plausible that Cas9 cannot fully operate around all chromatin structures. Indeed, recent evidence indicates that chromatin influences Cas9 binding by limiting the accessibility of the target site [10,18,22–25]. Cas9 takes longer to scan for the target sites buried in heterochromatin, whereas targets located in euchromatin are more accessible, and thus easier to locate [24]. However, heterochromatin does not entirely prevent Cas9 from binding to potential target sites and despite binding, cleavage does not necessarily occur [22,24]. Target site accessibility is reflected in the tendency of Cas9 to act on secondary targets, so it plays an important role when designing effective sgRNAs with maximum efficiency and a minimal number of off-targets [10,17,18]. If the intended target is buried in heterochromatin, it is more probable that Cas9 binds to secondary targets and is more likely to find those in the exon regions in euchromatin [18]. Evidence supporting the involvement of chromatin accessibility in Cas9 binding has emerged in *in vitro* models, cell lines and in the zebrafish (*Danio rerio*) [10,17,23–26]. However, detailed understanding on which chromatin features contribute to chromatin accessibility this is still lacking.

Compared to cell lines, zebrafish can present additional challenges for genome editing. Compared to other vertebrates, the teleost specific genome duplication has resulted in multiple similar genes or pseudogenes and this can, in some instances, complicate the identification of unique targets for sgRNA. Secondly, to generate mutant zebrafish, the sgRNA and Cas9 are microinjected into the fertilized embryo, and mutagenesis occurs during the first hours of development [27]. Compared to cell lines, the fertilized, CRISPR-injected zygote presents a challenge for all mutagenesis techniques as it undergoes developmental and differentiation processes that require global changes in chromatin. Lastly, the first cell division in zebrafish takes place very rapidly (40 minutes after fertilization), when compared to the cell divisions for example in mice (reaching E1.5 at 24 hours post fertilization, hpf). Mutagenesis occuring after this first cell division may more likely lead to mosaicism.

During development, the chromatin landscape is under constant change in order to enable coordinated growth and differentiation [28–30]. The zygote is supported by the available maternal transcripts and the zygotic genome remains transcriptionally inactive until the maternal to zygotic genome activation (MZT) at the mid blastula transition (MBT) [31]. Our current understanding of zygotic chromatin is limited, but it has been shown that a specific histone modification pre-patterning marks developmentally active and inactive genes during development [32]. The nuclease accessibility of the developing, chromatin-packed genome of embryos remains poorly understood. Previously, it was observed that chromatin does not influence CRISPR-Cas9 targeting in zebrafish embryos in an MNase assay (Micrococcal nuclease assay), but later ATAC-seq (Assay for Transposase-Accessible Chromatin using sequencing) results suggested that CRISPR-Cas9 is more likely to be successful when targeting open chromatin [9,17]. More information on the influence of chromatin on CRISPR-Cas9 mutagenesis in model organisms is needed in order to improve the efficiency of genome engineering methodologies.

In this study, we observed discrepancies between the *in vitro* and *in vivo* activities of sgRNAs, and that selected sgRNAs differ for their onset of mutagenesis. We saw an association between successful mutagenesis and the transcript levels during early development. We looked further into the involvement of gene activation and chromatin in explaining the CRISPR-Cas9 mutagenesis efficiency in zebrafish embryos. Our results indicate that gene expression and chromatin openness are associated with the efficiency of CRISPR-Cas9 mutagenesis. However, we saw no association of mutagenesis efficiency with either exon methylation or histone H3 Lysine 4 trimethylation (H3K4me3) at promoters.

## Results

### Good *in vitro* activity of sgRNA does not assure *in vivo* efficacy

**Fig 1:**
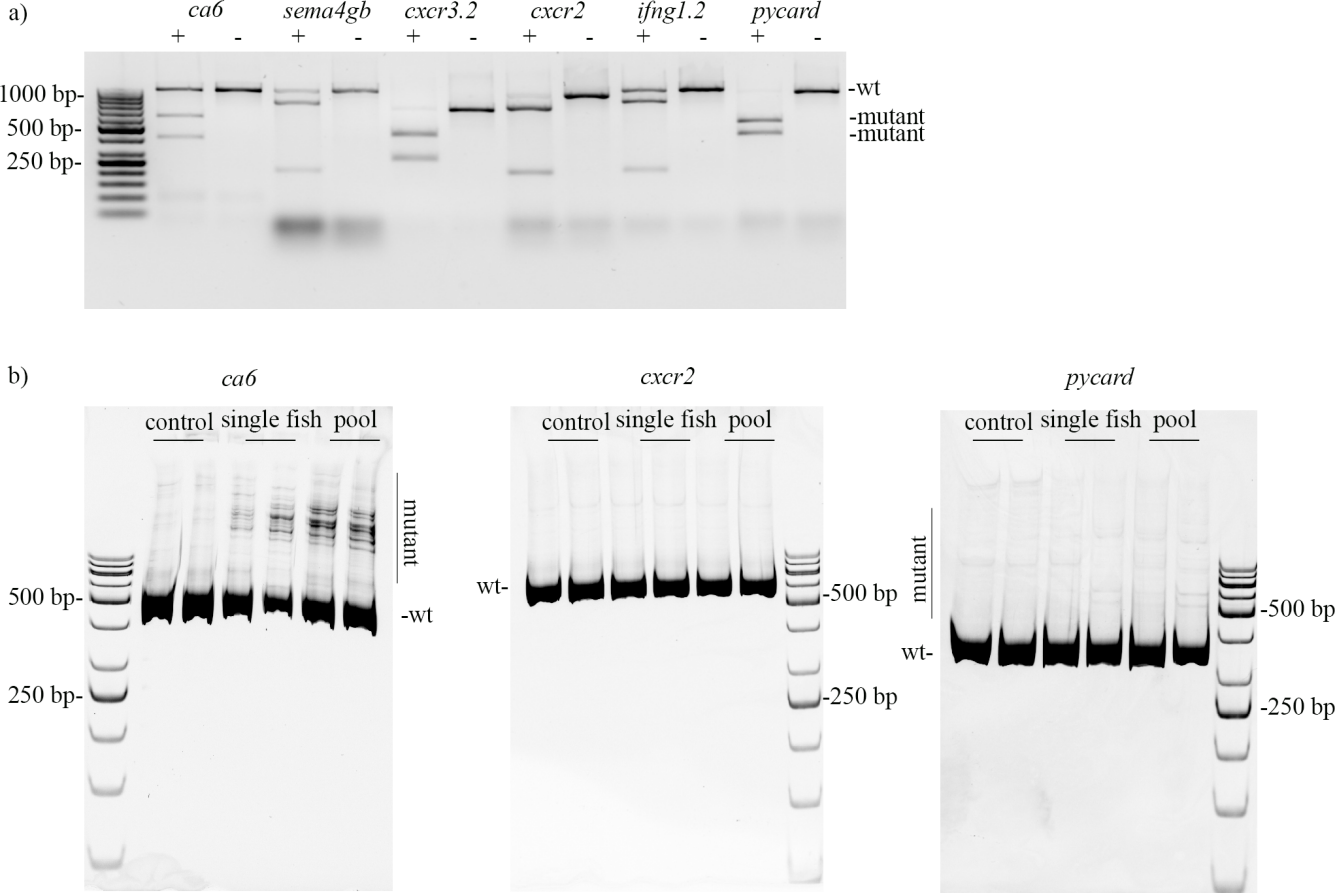
In vitro and in vivo CRISPR-Cas9 mutagenesis efficiencies do not correlate for all genes. a) An in vitro digestion assay shows that sgRNAs differ in their efficiencies. Below the gene name, + and - indicate the presence or absence of Cas9 protein in the reaction. On the right the wild type (wt) and the mutant products are indicated. b) The in vivo CRISPR-Cas9 mutagenesis visualized for ca6, cxcr2 and pycard with a heteroduplex mobility assay, with the wild type (wt) and the mutant products indicated. 5 embryos were collected per sample at 8hpf.

Analyzing the efficacy of different sgRNAs *in vivo* is laborious. To improve the screening for efficient sgRNAs, *in vitro* digestion of the target sequence can be used. We analyzed the mutagenesis activity of six sgRNAs first *in vitro* and then selected three for analysis *in vivo*. As shown in Fig 1, some sgRNAs with good *in vitro* efficiency presented low or no *in vivo* activity. This suggests that factors present *in vivo* prevent Cas9 from acting on its target site. Importantly, *cxcr2* had neither detectable gene expression nor mutagenesis efficiency, whereas the genes permissive for mutagenesis (*pycard*, *ca6*) showed early expression (Fig 1, S1 Fig). This led us to hypothesize that the onset and the level of gene expression could influence the CRISPR-Cas9 mutagenesis. The corresponding results using the T7 Endonuclease I assay are displayed in S2 Fig. In our hands the T7 Endonuclease I assay has a lower resolution compared to the heteroduplex mobility assay, especially with sgRNAs of lower efficiency. On the other hand, the T7 Endonuclease I assay can be readily used for quantitation of mutagenesis efficiency, especially with sgRNAs of higher efficiency.

### The onset of mutagenesis differs between sgRNAs *in vivo*

**Fig 2:**
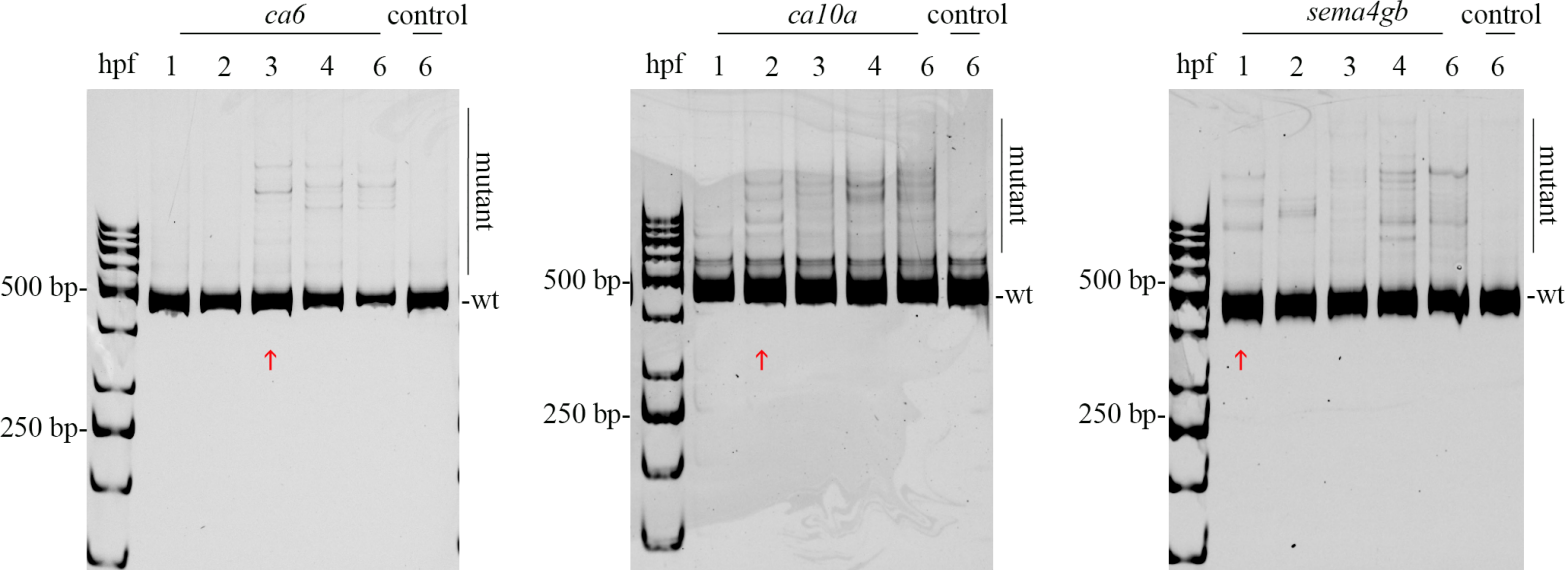
Onset of mutagenesis differs between sgRNAs. Heteroduplex mobility assay to demonstrate the onset of mutagenesis using high efficiency guide RNAs targeting three different genes with different gene expression patterns in early development. Embryos were collected at timepoints 1, 2, 3, 4, 6hpf (15–20 embryos per group). The gene name above the gel image indicates CRISPR-Cas9 injected embryos and control indicates uninjected controls. The legend on the side indicates the positions of wt (wild type) and mutant bands in the gel. Red arrows indicate the point at which first mutations can be detected.

As we saw a discrepancy between *in vivo* and *in vitro* mutagenesis efficiencies for some sgRNAs, we next analyzed whether the onset of mutagenesis correlates with the onset of gene expression. To avoid the delay of mRNA transcription for Cas9 activity, we used a ready Cas9 protein in our experiments with appropriate preincubation step to allow the sgRNA to complex with Cas9. Three of our functional sgRNAs were chosen for the analysis. The sgRNAs targeting *ca10a*, *sema4gb*, or *ca6* were co-injected with the Cas9 protein into the 1-cell stage embryo and the onset of mutagenesis was analyzed using both a heteroduplex mobility assay and a T7 Endonuclease I mutation detection assays. As shown in Fig 2 using the heteroduplex mobility assay, the first mutations become detectable as soon as 1hpf for *ca10a* and *sema4gb*, whereas the first mutations for *ca6* appeared at 3hpf (Fig 2). These results indicate that the onset of mutagenesis differs depending on the sgRNAs in zebrafish embryos. Based on these results, we analyzed the relationship of early gene expression and mutagenesis efficiency in more detail with all our sgRNAs. We were able to detect mutagenesis activity at 1hpf (roughly corresponding to 4-cell stage).

### Likelihood of successful mutagenesis in relation to the expression level of the target gene in zebrafish embryos

Altogether, we have designed 86 sgRNAs using the crispr.mit.edu, ChopChop (V1 and V2) and CRISPRscan softwares [9,14,15]. Of these sgRNAs, 30% showed detectable *in vivo* activity (S1 Table). As GC-content (%) has been suggested to influence the effectiveness of CRISPR-Cas9 mutagenesis, we analyzed the GC-content of our sgRNAs (S1 Table) [18]. The GC-content of our functional sgRNAs was found to be similar (Mann-Whitney U-test; p-value 0.452) to that of the non-functional sgRNAs.

**Fig 3:**
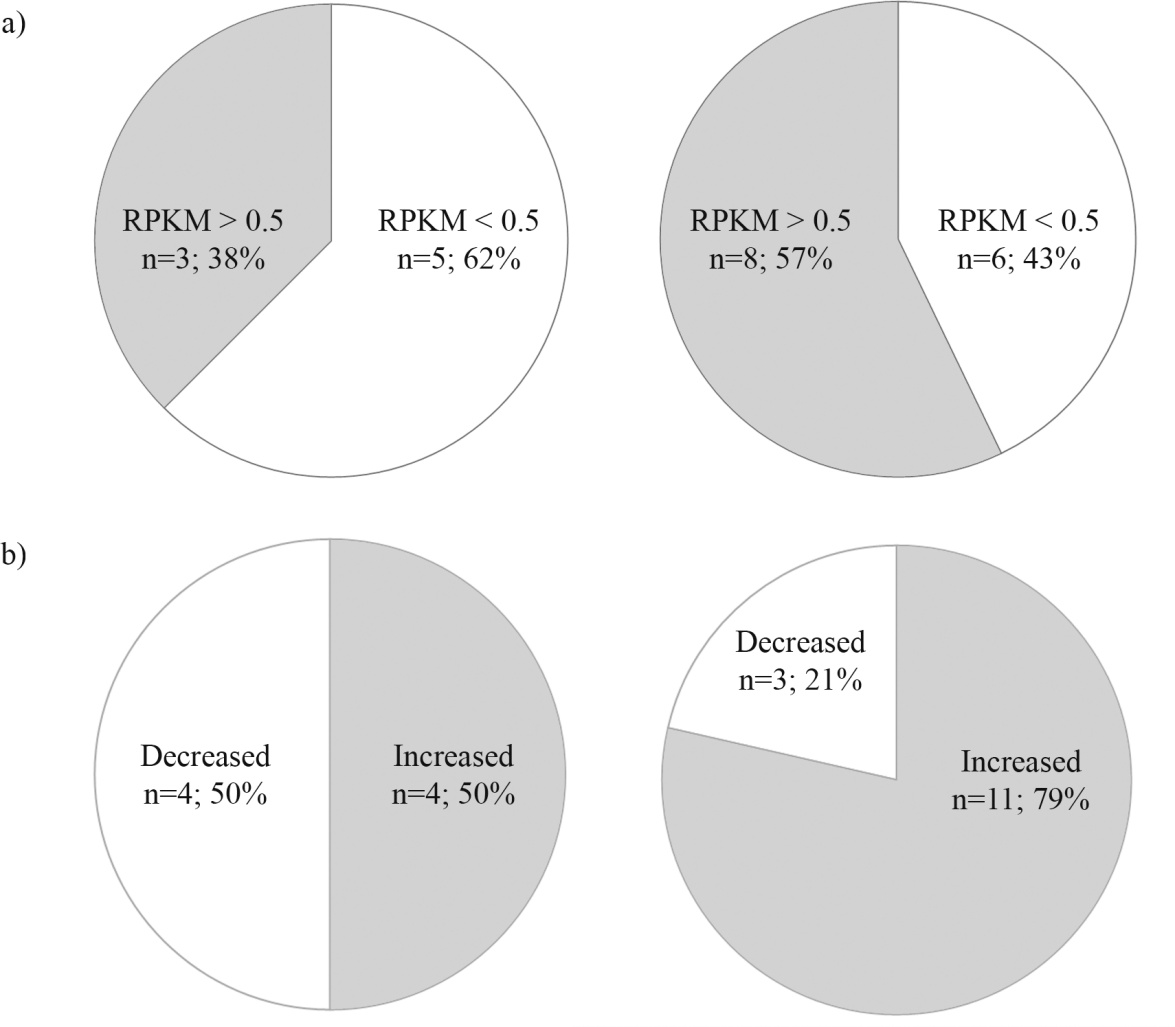
Relationship of transcript levels in early development and low mutagenesis efficiency. Pie charts of the RNA-seq data corresponding to graphs in S1 Fig. a) Number of transcripts for the genes resistant to (left) or permissive (right) for mutagenesis between the oblong sphere and the 15-somite stage (Fischer’s exact test; not significant). 0.5 RPKM (Reads per Kilobase of transcript per Million mapped reads) was used as a limit for low expression. b) The number of genes resistant (left) or permissive (right) for CRISPR-Cas9 mutagenesis in which the number of transcripts is increased or decreased between the oblong sphere-stage and 50% epiboly (around the MZT) (Fischer’s exact test; not significant).

When we compared the expression of the genes that we were able to mutate to those we were not, the genes resistant for mutagenesis more often had a very low expression level (Fig 3). However, the difference did not reach statistical significance (Fischer’s exact test; not significant). Moreover, a majority of genes (79%) permissive for mutagenesis underwent an increase in the number of transcripts around the MZT (identified here as a positive change in the number of transcripts between the oblong sphere stage and 50% epiboly). This occurred more often than in the genes resistant to mutagenesis (50%). However, this observation was not statistically significant (Fischer’s exact test) (Fig 3). To examine whether the lack of statistical significance was due to a type two error, we decided to determine whether there is a correlation between target gene expression and mutagenesis efficiency using larger datasets.

### Mutagenesis efficiency correlates with gene expression and chromatin accessibility in zebrafish embryos

In searching for factors that would explain the poor *in vivo* activity of some sgRNAs, we investigated available open access datasets. As genes with low expression values tended to be more difficult to mutate in our setting (Fig 3), we analyzed the association between expression levels and mutagenesis efficiency in greater depth, using large datasets in order to avoid type 2 error. We obtained CRISPR-Cas9 sgRNA efficiency data from CRISPRz database for all analyses [33]. We used open access RNA-seq data (E-GEOD-45706) for our primary analysis of the correlation between CRISPR-Cas9 mutagenesis and gene expression [33,34]. We found significant correlations in early development (between 64-cell stage and 36hpf), at the oblong sphere stage (3.66hpf, Spearman correlation 0.227; p-value 0.001) and at 36hpf (Spearman correlation 0.230; p-value 0.001). A strong correlation was observed at the oblong sphere stage which occurs shortly after MBT, around the time of zygotic genome activation. These results suggest that transcriptional activity influences CRISPR-Cas9 mutagenesis at early development (Table 1).

**Table 1:** Correlation between mutagenesis efficiency and gene expression at different developmental stages during early development. n = 209.

Developmental stage	Spearman correlation	p-value
64-cell	0.190	0.006*
oblong-sphere	0.227	0.001*
50%-epiboly	0.187	0.007*
15-somite	0.210	0.002*
36hpf	0.230	0.001*
48hpf	0.182	0.008*
60hpf	0.188	0.006*
72hpf	0.131	0.058

As methylation is known to correlate with transcriptional repression, we used zebrafish exon methylation data to analyze whether there is any correlation between exon methylation and the success of CRISPR-Cas9 mutagenesis [33,35]. As is shown in Table 2, there was no significant correlation between exon methylation and CRISPR-Cas9 mutagenesis efficiency at the 1-cell stage or at MBT (Table 2). Similarly, using open access data on embryonic histone methylation, we analyzed whether there is a correlation of H3K4me3 at promoters with CRISPR-Cas9 mutagenesis efficiency. As shown in Table 2, there seemed to be a correlation but this did not reach statistical significance (Spearman correlation 0.263; p-value = 0.074) [33,36].

**Table 2:** Correlation between mutagenesis efficiency and chromatin features at different developmental stages and timepoints.

Chromatin feature	n	Developmental stage/Timepoint	Spearman correlation	p-value
Exon methylation	263	1-cell	0.115	0.063
		Mid blastula transition	0.107	0.084
H3K4me3	47	75–80% epiboly	0.263	0.074
Chromatin accessibility	263	4hpf	0.182	0.003*

ATAC-sequencing is a recent next generation sequencing method, which can be used to directly analyze chromatin accessibility. Open access ATAC-seq data for the zebrafish embryo is available at the 4hpf timepoint [37]. We compared mutagenesis efficiency data with ATAC-seq data at transcription start sites for a total of 263 genes. We discovered a significant, albeit rather weak correlation, indicating that chromatin accessibility appears to be one of the factors that explain the efficiency of CRISPR-Cas9 mutagenesis in zebrafish embryos (Table 2).

## Discussion

In this study, we found discrepancies between the *in vitro* and *in vivo* efficiencies of some sgRNAs. These discrepancies suggested the presence of cellular factors which limit mutagenesis, and encouraged us to analyze chromatin involvement in more detail at the transcriptomic and epigenomic levels. Because the transcript counts of the early embryo can be masked by the presence of maternal transcripts, it is difficult to establish the exact relationship between gene expression and mutagenesis efficiency [38]. However, we found weak but significant correlations of gene expression with mutagenesis efficiency during early development, with the strongest correlation at the oblong sphere stage (3.66hpf, Spearman correlation 0.227; p-value 0.001) and later at 36hpf (Spearman correlation 0.230; p-value 0.001). The correlation at the oblong sphere stage suggests that genes which become active at the MZT are more accessible for Cas9 and hence undergo more efficient mutagenesis.

As chromatin structure is complex, its effect on target site accessibility has to be determined for each structural level, starting with direct modifications to DNA bases, continuing with analysis of histone modifications signaling for open chromatin, and ending with analysis of chromatin accessibility. A detailed analysis is required in order to understand how CRISPR-Cas9 mutagenesis activity could be manipulated at molecular level using for example chemical inhibitors of histone deacetylase activity. DNA methylation is known to mark transcriptional inactivity and recruit modified histones at the exons [39]. In our study, exon methylation was not found to significantly influence the activity of mutagenesis in zebrafish embryos. In confirmation, it has previously been suggested that Cas9 can act independently from DNA methylation in cell lines, and that, in general, most protein-DNA interactions are independent of DNA methylation [7,40]. If DNA methylation is not a limiting factor, we hypothesized that mutagenesis might correlate with higher order structures, specifically histone modifications. Various histone modifications mediate transcriptional activation and repression, and form nucleosome structures, which bind chromatin into an inactive heterochromatin state. H3K4me3 is a well known modification occurring in early development [41]. The most strongly suggestive, albeit not significant, correlation between experimental data and CRISPR-Cas9 mutagenesis efficiency was found with H3K4me3 data (Spearman correlation 0.263, p-value 0.07) [32]. This was expected, given the association with transcriptional activity at early developmental stages. As mutagenesis can already be detected at 1hpf it is possible that we fail to see a stronger correlation because the inspected timepoint is late and the histone landscape at 75–80% epiboly is dissimilar to that which is present before the MBT. In addition, if data from multiple timepoints would be available it would provide a more comprehensive view to opening of local chromatin structures. Also, observing only H3K4me3 signals might not accurately reflect the chromatin state in early embryos, as there are also other histone marks for open and closed chromatin, including H3K9me3 and H3K27me3 as well as H3K27ac at promoters [28,32,42]. A wider scale analysis of histone modifications could provide more insight into the association of CRISPR-Cas9 efficiency with histone landscape.

A higher order structure above the histone landscape is shaped by modified histones organizing into nucleosomes. Nucleosome occupancy, breathing and remodeling have previously been found to affect the cleavage activity of Cas9 and consequently, CRISPR-Cas9 mutagenesis is more successful when targeting the sequences depleted in nucleosomes [25,26,43]. The position of the PAM-sequence relative to nucleosomes has been found to be a key determinant of the Cas9 endonuclease activity *in vitro* but not in zebrafish [17,23]. Nucleosomes affect chromatin accessibility, which can be measured using ATAC-seq [44]. This state-of-the-art method has been used for identification of accessible chromatin regions during early development [37]. Using the publicly available data, we found a weak but significant correlation between chromatin accessibility and mutagenesis efficiency at the MBT, indicating that chromatin influences the efficiency of CRISPR-Cas9 mutagenesis in zebrafish embryos, even though it is not the sole defining factor (Table 2) [37]. Our results are in line with those by others [17] with different analysis method and dataset. Moreover, our results suggest CRISPR-Cas9 mutagenesis efficiency to be independent of exon methylation and H3K4me3 at promoters.

Deciphering the effect of developmental chromatin on the activity of CRISPR-Cas9 mutagenesis model organisms ultimately leads us to an unanswered question about the regulation of zygotic genome activation and the signals that regulate this event at early stages before the MTZ [45]. The genome remains in a transcriptionally inactive state before the MZT, and it is likely that this inactive chromatin also limits the access of mutagenesis reagents such as Cas9. It is also likely that Cas9 can gain access during replication, and at sites that contain more permissive histone modifications or are depleted in nucleosomes, but only with limited efficacy. With further cell divisions, chromatin repressive signals then become diluted, leading to chromatin opening at the MZT and initiation of transcription [45]. Despite the biological significance of the MBT and MZT, we were already able to see mutagenesis taking place at 1hpf for some genes, so we propose that (when designing CRISPR-Cas9 mutagenesis strategies) chromatin structure should be taken into account at a very early timepoint (Fig 2).

Several studies have looked into the correlation of *in silico* predictions and *in vivo* activity of sgRNAs and found that CRISPR-sgRNA design tools often fail to accurately predict sgRNA activity [11,20,25]. Moreover, it has been observed, that the *in silico* predictions which are efficient for model organisms are not efficient for cell line based assays and *vice versa* [11]. As Haeussler *et al*. (2016) observed, CRISPR-Cas9 efficiency in mice is better predicted by the algorithms that have been trained on zebrafish experimental data, than by cell line based algorithms. It is logical to assume this is at least in part due to the fact that mice and zebrafish undergo similar, conserved developmental dynamics at the transcriptomic and epigenomic level (at the time when CRISPR-mutagenesis is taking place), and target site accessibility is largely defined by early chromatin. Thankfully, design tools, which also take into account target site accessibility, have recently become available [11,14,16,17]. Detailed analysis is required to pinpoint which are the most important chromatin structures impacting CRISPR-Cas9 activity. With a better understanding of these, we will hopefully achieve improvements in predictions for experimental design especially in the *in vivo* models. Eventually, it might be possible to modify local chromatin to increase target site accessibility and simultaneously decrease the likelihood of off-target binding. Our results confirm the involvement of chromatin in defining CRISPR-Cas9 mutagenesis efficiency in a vertebrate model *in vivo*.

## Materials and methods

### Zebrafish maintenance

Wild type AB fish were maintained in a flow-through system with a light/dark cycle of 14h/10h according to the standard procedure. Embryos and larvae were grown in an incubator (28.5°C) in embryonic medium/E3 water (5mM NaCl, 0.17mM KCl, 0.33mM CaCl_2_, 0.33mM MgSO_4_, and 10–15% Methylene Blue).

### Ethics statement and data availability

All experiments were carried out in accordance with the EU-directive 2010/ 63/EU on the protection of animals used for scientific purposes, and with the Finnish Act on the Protection of Animals Used for Scientific or Educational Purposes (497/2013) and the Government Decree on the Protection of Animals Used for Scientific or Educational Purposes (564/2013). We have only used zebrafish prior to their independently feeding larval stages in this study, which thus do not require animal permits. Permit for the zebrafish housing and maintenance for the facility at the University of Tampere is ESAVI/10079/04.10.06/2015.

The computational data analysed in this study were collected from open access sources, as detailed in the appropriate sections.

### Design and production of sgRNAs for CRISPR/Cas9 mediated genome editing

Target sequences (S1 Table) for sgRNA design were chosen using the online based CRISPR design tool (http://crispr.mit.edu/), ChopChop.V1 or V2 [14,15] or CRISPRscan [9]. Target site uniqueness was verified with the NCBI BLAST analysis against the zebrafish genome (GRCz10). sgRNAs were produced as described previously [46]. Briefly, the sgRNA oligo (Sigma-Aldrich) and the T7 promoter site oligo (S1 and S2 Tables) (Sigma-Aldrich) were annealed and *in vitro* transcribed using the MEGAshortscript T7 Transcription Kit (Ambion Life Technologies, CA, USA). The integrity and size of the produced sgRNAs were analyzed with gel electrophoresis (1% agarose in Tris-acetate-EDTA, TAE). The concentration of the sgRNAs was measured with the Qubit® RNA BR Assay kit (Thermo Fisher Scientific, MA USA 02451) and Nanodrop 2000 (Thermo Fischer Scientific).

### sgRNA and Cas9 microinjection and genomic DNA extraction

The sgRNAs and the Cas9 protein (ToolGen Inc., Seoul, South Korea) were co-injected into one-cell stage zebrafish embryos with a micro injector (PV830 Pneumatic PicoPump, World Precision Instruments) under a Nikon microscope (SMZ645), using borosilicate needles prepared with a Flaming/Brown micropipette puller. Needles were calibrated by injecting solution into a halocarbon oil droplet to achieve a diameter of 12μm (approximately 1nl). The embryos were aligned on 1.2% agarose E3 water plates prior to the injection. An injection solution containing 130ng/μl sgRNA and 250ng/μl of the Cas9 protein in nuclease-free water was incubated 37°C 15min. Rhodamine dextran was added to the solution for the visualization of the injections under a Zeiss Lumar V12 fluorescence microscope. To analyze the onset of the mutagenesis 10–20 CRISPR-Cas9 injected embryos were collected and frozen in liquid nitrogen for DNA extractions at 1, 2, 3, 4, 6hpf (hours post fertilization). To analyze the *in vivo* mutagenesis efficiency, 5 embryos were collectedat 8hpf and immediately frozen in liquid nitrogen. For DNA extraction, the embryos were lysed 4h 55°C in lysis buffer (10mM Tris pH 8,2, 10mM EDTA, 200mM NaCl, 0.5% SDS, 200μg/ml Proteinase K). DNA was precipitated 1h -20°C using two volumes of ethanol. DNA was then pelleted by centrifuging 16,000g 10min. The pellet was washed with 200μl of 70% ethanol before resuspending in 200μl of water. A purification step with phenol-chloroform was performed after treatment with 15u of RNase A (Thermo Fischer Scientific) per 100μl of sample, 1h 37°C.

### Heteroduplex mobility assay

Targeted loci were amplified from the genomic DNA by PCR using the Maxima Hot Start DNA polymerase (Thermo Fischer Scientific) according to the manufacturer’s instructions. The PCR primers (S3 Table) were designed to anneal upstream and downstream of the expected cutting site. The PCR product was purified using Exo I and FastAP (Thermo Fischer Scientific) treatment 15min 37°C, then 15min 85°C. 10μl of the purified PCR product was annealed in a reaction containing 1x NEBuffer 2 (New England Biolabs, MA, USA) and was run on a 10% polyacrylamide gel. The gel was stained with GelRed (Bitium Inc., Fremont, CA).

### T7 Endonuclease I mutation detection assay

After purifying and annealing the PCR amplified locus, 10μl of this product was incubated 30min 37°C with 6 units of T7 Endonuclease I (New England Biolabs). The obtained products were separated on a 2.0% agarose TAE gel. The gel was stained with GelRed. The band sizes were compared to control samples.

### *In vitro* digestion of DNA with the Cas9-gRNA complex

To test the *in vitro* cutting potential, equimolar amounts of the Cas9 protein (ToolGen Inc.) and sgRNA were pre-incubated 15min 37°C in NEB 3 Buffer (New England Biolabs) and 1% Bovine serum albumin (Sigma Aldrich). For the template, a 850–1,200bp site around the target was amplified using Maxima Hot Start DNA polymerase according to the manufacturer’s instructions. The template was then purified (GeneJET PCR Purification kit, Thermo Fischer Scientific). The template was then added to a final 10:10:1 ratio (Cas9:sgRNA:template PCR product). The reaction mix was incubated 3h 28°C as this is the temperature at which zebrafish embryos are maintained. After this, we incubated the sample with 300U of Proteinase K 37°C 10min to release the Cas9. Proteinase K was inactivated by incubation 65°C 10min. Samples were run on a 1% agarose TAE gel to analyze the cutting efficiency.

### Gene expression analysis of CRISPR targeted genes

The CRISPRz database contains a list of 1,398 validated zebrafish sgRNAs collected from various published resources [33]. In addition to sgRNA sequences, the associated mutagenesis efficiencies have been recorded in 325 unique zebrafish genes. We compared these mutagenesis efficiencies, from somatic cells, with a publicly available RNA-seq expression dataset housed in the ArrayExpress database [47]. The dataset (ArrayExpress E-GEOD-45706: https://www.ebi.ac.uk/arrayexpress/experiments/E-GEOD-45706) consists of RNA-seq data performed for samples from multiple stages of zebrafish development: 64-cell, oblong-sphere, 50%-epiboly, 15-somite, 36hpf, 48hpf, 60hpf and 72hpf (and 1 week, excluded from this analysis). Using the Stats package of the SciPy library, we performed Spearman rank correlation analyses of expression data for each sample in each ArrayExpress RNA-seq dataset; using the expression values for genes with available mutagenesis data for somatic cells in CRISPRz [48].

### Histone modification in zebrafish promoters

The ArrayExpress dataset E-GEOD-4863 (https://www.ebi.ac.uk/arrayexpress/experiments/E-GEOD-4863/) is based on custom microarrays for the identification of ChIP binding sites of antibodies against the H3K4me3 in the promoters of zebrafish genes [36]. From the microarray datasets, the log of the median values of the 60-mer probes were summed for each gene, averaged, and then paired with CRISPRz mutagenesis values. Subsequently, these paired values were used to perform the Spearman rank correlation analysis.

### Exon methylation analysis of zebrafish genes

McGaughey *et al*. showed that exon methylation was a better indication of mRNA expression than promoter methylation [35]. Their genome-wide ChIP-seq analysis of whole embryo zebrafish DNA methylation is available as an ArrayExpress dataset E-GEOD-52110 (https://www.ebi.ac.uk/arrayexpress/experiments/E-GEOD-52110/) at the 1-cell stage and at MBT. We first translated all ChIP-seq peaks from Zv9 genome coordinates to GRCz10 coordinates and then mapped them to exons annotated in the GRCz10 genome. A summation of all ChIP-seq peaks which overlapped exons was calculated for each gene, this sum was divided by the total length of the gene’s exons to generate a methylation coefficient. The methylation coefficients were then combined with mutagenesis data to compute Spearman rank correlations for each timepoint.

### ATAC-seq analysis of zebrafish transcriptional units

ATAC-seq is a powerful method for identifying regions of accessible chromatin and it can be used to generate nucleotide resolution mapping of the hyperactive Tn5 transposase binding sites in the genome. An ATAC-seq analysis of 4hpf zebrafish has been previously completed and is available as an ArrayExpress dataset E-GEOD-74231 (https://www.ebi.ac.uk/arrayexpress/experiments/E-GEOD-74231/) [37]. SRR2747531 was downloaded from the NCBI Sequence Read Archive [37]. Reads were inspected using Fastqc version 0.11.5 and deemed to be of good quality and no further quality filtering or trimming was performed [49]. Subsequently, reads were aligned with Bowtie2 version 2.3.2 [50] using the parameter—very-sensitive-local against the Ensembl Zebrafish reference genome GRCz10. Alignments were filtered and sorted using samtools version 1.4 with the parameter -q 20. Duplicates were removed using Picard Markduplicates version 2.6.0 with the parameters REMOVE_DUPLICATES = TRUE VALIDATION_STRINGENCY = LENIENT [51]. As the alignment was performed against recent reference it was necessary to perform peak- calling independently of the original paper [37]. Furthermore, due to advances in peak calling softwares, peak calling was performed with macs2 version 2.1.1 using the parameters—nomodel—shift -100—extsize 200 -q 0.05 –broad [52]. Transcription start sites (TSSs) for all transcripts annotated in the GRCz10 genome were pooled for each gene. TSSs within 500nt were clustered as a single transcriptional unit. Of the total 22,152 zebrafish genes, 18,687 had a single transcript. Subsequent clustering created single transcriptional units in 2,233 of the remaining 3,465 genes with more than one annotated transcription start site. For clustered TSSs, the midpoint was used as the representative TSS. For each TSS, a +/- 1,000nt region was used to associate ATAC-seq peaks from the E-GEOD-74231 dataset. For each of these regions, an ATAC-seq coefficient was generated by summation of the product of ATAC-seq signal value by total overlap with the TSS region, divided by the length of the region (2,000nt). In cases where after clustering a gene still had more than one TSS ATAC-seq peak, a correlation was performed for all TSS regions and then averaged. Subsequently, all zebrafish genes possessed a single ATAC-seq coefficient. These were then combined with the mutagenesis data from the CRISPRz database in order to compute the Spearman rank correlation.

## Supporting information

S1 Table sgRNA target site sequences for each genomic target. sgRNAs used in the experiments in this paper are indicated by a * after the gene name. Functional (Yes/No) indicates observed in vivo activity.
(DOCX)

S2 Table sgRNA template sequence. The extra 3’ guanines (G/GG) were used if target sequence has one or two 5’ guanines. N- indicates the position of the target sequence.
(DOCX)

S3 Table Primers used in T7 Endonuclease I assay (T7EI), Heteroduplex mobility assay (HMA) and *In vitro* Digestion Assay (IVDA). (DOCX)

S1 Fig The relationship of mutagenesis efficiency and transcript level. The graph a) presents the expression of the genes resistant to CRISPR-Cas9 mutagenesis, at the early stages of development. The graph b) presents the genes that were successfully mutated with CRISPR-Cas9. 2–10 sgRNAs have been used for mutagenesis. RPKM, Reads per Kilobase of transcript per Million mapped reads. All sgRNA sequences have been given in **S1 Table**.
(TIF)

S2 Fig T7 endonuclease I assay results corresponding to Fig 1. The *in vivo* CRISPR-Cas9 mutagenesis efficiencies for selected genes estimated with the T7EI assay for *ca6*, *cxcr2* and *pycard*. 5 embryos were collected per sample at 8hpf. Black arrows indicate the mutated cleavage products for *ca6*.
(TIF)
