# Supplementary material for: Chromatin accessibility is associated with CRISPR-Cas9 efficiency in the zebrafish (Danio rerio)
Source: PLoS One. 2018 Apr 23;13(4):e0196238. doi: 10.1371/journal.pone.0196238 (PMC5912780; doi:10.1371/journal.pone.0196238)
Supplement: S1 Table — sgRNAs used in the experiments in this paper are indicated by a * after the gene name. Functional (Yes/No) indicates observed in vivo activity. (DOCX) [file pone.0196238.s001.docx]

**S1 Table. sgRNA target site sequences for each genomic target. sgRNAs used in the experiments in this paper are indicated by a * after the gene name. Functional (Yes/No) indicates observed in vivo activity.**

| Ensembl ID | Functional | Gene name | sgRNA | Target sequence | PAM |
| --- | --- | --- | --- | --- | --- |
| ENSDARG00000016918 | No | *ace2* | 1 | GAGGCCAAGGCCAAAAGC | AGG |
| ENSDARG00000016918 | No | *ace2* | 2 | GATATCTGTGTTGTAGGCCC | AGG |
| ENSDARG00000052644 | No | *ca10a* | 1 | TCCACCCAAAATCCATGA | GGG |
| ENSDARG00000052644 | No | *ca10a* | 2 | TACAAAGAAGTTGTTCAG | GGG |
| ENSDARG00000052644 | Yes | *ca10a** | 3 | ACTGAGGCTCAACACTGG | CGG |
| ENSDARG00000009568 | Yes | *si:dkey-15h19.2* | 1 | AACGAACTCCCAAACGTG | CGG |
| ENSDARG00000009568 | No | *si:dkey-15h19.2* | 2 | TTGGGAAGAGACAGTCGC | CGG |
| ENSDARG00000056499 | Yes | *ca6* | 1 | GGTTATGAAGACATCCG | CGG |
| ENSDARG00000056499 | Yes | *ca6** | 2 | GGGGAGGCTGGGACTTGG | AGG |
| ENSDARG00000070873 | Yes | *ccl25b* | 1 | GATACATGCAAGCCAACAGC | AGG |
| ENSDARG00000070873 | No | *ccl25b* | 2 | GAGTGCAGCTAACAGA | CGG |
| ENSDARG00000079829 | No | *si:ch211-207g17.2* | 1 | CCATGCAACAATGGCAAC | GGG |
| ENSDARG00000079829 | No | *si:ch211-207g17.2* | 2 | TTGTCCCATTATTAGCAT | TGG |
| ENSDARG00000014522 | No | *cdh6* | 1 | GCTGAGACGTTCAAAGCG | AGG |
| ENSDARG00000014522 | No | *cdh6* | 2 | GCCCTTTCAAGAGGGC | CGG |
| ENSDARG00000008674 | Yes | *chrnb2a* | 1 | GTGGGATCCAAATGAGTATG | AGG |
| ENSDARG00000008674 | Yes | *chrnb2a* | 2 | GGAATGACTACAGACTGGTG | TGG |
| ENSDARG00000008674 | No | *chrnb2a* | 3 | ATCATGACAACAAATGTC | TGG |
| ENSDARG00000008674 | No | *chrnb2a* | 4 | CAGTCAATAAGAGTCAAC | AGG |
| ENSDARG00000008674 | No | *chrnb2a* | 5 | AAGCTTATTATAACGCTC | AGG |
| ENSDARG00000008674 | No | *chrnb2a* | 6 | GTCTCTTGCTGCTGATGCTG | AGG |
| ENSDARG00000008674 | No | *chrnb2a* | 7 | GTTGATCAAAAGGGAAGTTG | CGG |
| ENSDARG00000008674 | No | *chrnb2a* | 8 | TGCGGTCGTAGGTCCAAGAG | CGG |
| ENSDARG00000017790 | Yes | *chrnb2b* | 1 | AGAAGACACTTCCATCAT | AGG |
| ENSDARG00000017790 | Yes | *chrnb2b* | 2 | GGATCTGCTCTACCTCGG | GGG |
| ENSDARG00000054975 | No | *cxcr2* | 1 | AGGTTCTTCACGGTCTCC | GGG |
| ENSDARG00000054975 | Yes | *cxcr2** | 2 | TTCTTCATGGACAACCGC | AGG |
| ENSDARG00000054975 | No | *cxcr2* | 3 | GGTGGCGTACCTCCACGC | CGG |
| ENSDARG00000054975 | No | *cxcr2* | 4 | GGCCAGTGACCGGCGTGG | AGG |
| ENSDARG00000041041 | Yes | *cxcr3.2** | 1 | TAGTGGGCACGAGATGTT | AGG |
| ENSDARG00000041041 | No | *cxcr3.2* | 2 | CTTCTCTGAAGCTCATCA | TGG |
| ENSDARG00000041041 | No | *cxcr3.2* | 3 | GTTTATCCTGGCGCTAGT | GGG |
| ENSDARG00000069018 | Yes | *cyp7a1a* | 1 | CCACTATGGCCCAAATGA | AGG |
| ENSDARG00000069018 | Yes | *cyp7a1a* | 2 | GGTAGGAGAATGGATCACAA | AGG |
| ENSDARG00000069018 | Yes | *cyp7a1a* | 3 | GCTGTCATCCGTCAAGGA | AGG |
| ENSDARG00000079850 | Yes | *dchs1b* | 1 | GCCAGGCGACTTGACCGAG | AGG |
| ENSDARG00000079850 | Yes | *dchs1b* | 2 | GGATTCTGCATGTAGTGGC | GGG |
| ENSDARG00000079850 | Yes | *dchs1b* | 3 | GATCTGCACCAGCACGGCTC | TGG |
| ENSDARG00000079850 | No | *dchs1b* | 4 | GGAGATTCTCGCAACATAT | TGG |
| ENSDARG00000077540 | No | *f2rl1.2* | 1 | CCCAGCTGCCATTTACAT | GGG |
| ENSDARG00000077540 | No | *f2rl1.2* | 2 | ATAACTGGACGTATGGCG | AGG |
| ENSDARG00000077540 | No | *f2rl1.2* | 3 | TCAGTGGGCTTCTTCTAC | GGG |
| ENSDARG00000077540 | No | *f2rl1.2* | 4 | GGCGGTGTCCGAGAGCTAC | AGG |
| ENSDARG00000077540 | No | *f2rl1.2* | 5 | GGGTTGCCAACCAACGCAA | TGG |

**S1 Table. Continued. Guide RNA target site sequences for each genomic target. sgRNAs used in the experiments in this paper are indicated by a * after the gene name. Functional (Yes/No) indicates observed in vivo activity.**

| **Ensembl ID** | **Functional** | ***Gene name*** | **sgRNA** | **Target sequence** | **PAM** |
| --- | --- | --- | --- | --- | --- |
| ENSDARG00000077540 | No | *f2rl1.2* | 6 | GAATAACTGGACGTATGGCG | AGG |
| ENSDARG00000070448 | Yes | *grk4* | 1 | GGCCAACACCGTGCTGCTGA | AGG |
| ENSDARG00000070448 | Yes | *grk4* | 2 | GCGGAGGGAAGCGTAATGGA | CGG |
| ENSDARG00000070448 | No | *grk4* | 3 | GGCGCTGTATAGAGTTCC | TGG |
| ENSDARG00000070448 | Yes | *grk4* | 4 | GCGTCTCTCCTCTTCTCATC | CGG |
| ENSDARG00000045671 | Yes | *ifng1-1* | 1 | AGCGCATACAGATTTCGA | CGG |
| ENSDARG00000045671 | No | *ifng1-1* | 2 | GCTTGCAAAGGATTGGGT | TGG |
| ENSDARG00000045671 | No | *ifng1-1* | 3 | ACACAGCCTGGCAAGTGC | AGG |
| ENSDARG00000024211 | No | *ifng1-2** | 1 | TGTTTGCTGTTTTCGGGA | TGG |
| ENSDARG00000024211 | Yes | *ifng1-2* | 2 | CTAGGTTCTCGGGCACAC | TGG |
| ENSDARG00000024211 | No | *ifng1-2* | 3 | AGGATTCGCAGGAAGATG | GGG |
| ENSDARG00000007534 | No | *itln1* | 1 | GTAGGGGTTTTACCTGT | AGG |
| ENSDARG00000007534 | No | *itln1* | 2 | GACATGAGCACCAACGGTGG | AGG |
| ENSDARG00000003523 | Yes | *itln3* | 1 | AGGTTGAGGAGCATCGCT | CGG |
| ENSDARG00000003523 | No | *itln3* | 2 | TGACATGACCACAGCCGG | AGG |
| ENSDARG00000003523 | No | *itln3* | 3 | CATCCTGACAACAGAGAG | CGG |
| ENSDARG00000010169 | No | *myd88* | 1 | GGCGGCAGACTGGAGGACAG | TGG |
| ENSDARG00000010169 | No | *myd88* | 2 | GATGGACTTCACGTACC | TGG |
| ENSDARG00000010169 | No | *myd88* | 3 | GTTACTGGAATCGCCTCA | TGG |
| ENSDARG00000089581 | No | *nhlrc2* | 1 | TCTAGCTGGCCCTGAATG | AGG |
| ENSDARG00000089581 | No | *nhlrc2* | 2 | GGGAGGATTTGATTGTAC | CGG |
| ENSDARG00000089581 | No | *nhlrc2* | 3 | TCAAAATAGACGAGAGGG | AGG |
| ENSDARG00000089581 | No | *nhlrc2* | 4 | TTCCTTCCTGAGTGACAG | CGG |
| ENSDARG00000089581 | No | *nhlrc2* | 5 | TAAAGAAGGCGGAGCATT | GGG |
| ENSDARG00000089581 | No | *nhlrc2* | 6 | GGAATGGTTAAACACAGA | CGG |
| ENSDARG00000089581 | No | *nhlrc2* | 7 | GTCCGCTGTCACTCAGGA | AGG |
| ENSDARG00000089581 | No | *nhlrc2* | 8 | GAGATGGAAATTTGTCCG | AGG |
| ENSDARG00000089581 | No | *nhlrc2* | 9 | CCAAGGGTAACATCCCA | GGG |
| ENSDARG00000089581 | No | *nhlrc2* | 10 | GCACTGATAAAGAAGG | CGG |
| ENSDARG00000104197 | Yes | *ptpn22* | 1 | GGCAGAGAACGGCTTTGCCG | GGG |
| ENSDARG00000104197 | No | *ptpn22* | 2 | GATGAAACGGCAGAGAA | CGG |
| ENSDARG00000040076 | No | *pycard** | 1 | TGCAGACTTTGTGACGCG | CGG |
| ENSDARG00000040076 | No | *pycard* | 2 | AACTGGGCGATCGGAGGC | AGG |
| ENSDARG00000040076 | No | *pycard* | 3 | CGTGTTCACATCAAAAGACG | CGG |
| ENSDARG00000104290 | No | *ripk2* | 1 | GCACTACATCAGCAAGGG | AGG |
| ENSDARG00000104290 | No | *ripk2* | 2 | GGGATCACCGGTAATGTGC | TGG |
| ENSDARG00000088143 | Yes | *sema4gb** | 1 | GATCCCACTAAGGGGTACAC | TGG |
| ENSDARG00000088143 | No | *sema4gb* | 2 | GAAGGCGTGAGTCCCGC | AGG |
| ENSDARG00000088143 | No | *sema4gb* | 3 | GTAAGCACAGCGGGGTCTGA | AGG |
| ENSDARG00000088143 | No | *sema4gb* | 4 | GAATGCTACAATCACGTG | CGG |
| ENSDARG00000007990 | Yes | *wt1b* | 1 | GCTGGAAGCAGGGCGCTC | AGG |
| ENSDARG00000007990 | No | *wt1b* | 2 | GGGTCCTCGTAAGGGTCTGG | GGG |
