# Supplementary material for: Chromatin accessibility is associated with CRISPR-Cas9 efficiency in the zebrafish (Danio rerio)
Source: PLoS One. 2018 Apr 23;13(4):e0196238. doi: 10.1371/journal.pone.0196238 (PMC5912780; doi:10.1371/journal.pone.0196238)
Supplement: S2 Table — The extra 3’ guanines (G/GG) were used if target sequence has one or two 5’ guanines. N- indicates the position of the target sequence. (DOCX) [file pone.0196238.s002.docx]

**S2 Table. sgRNA template sequence. The extra 3’ guanines (G/GG) were used if target sequence has one or two 5’ guanines. N- indicates the position of the target sequence.**

| 5’GCGTAATACGACTCACTATA(**G/GG/GGG)**NNNNNNNNNNNNNNNNNNGTTTTAGAGCTAGAAATAGCAAGTTAAAATAAGGCTAGTCCGTTATCAACTTGAAAAAGTGGCACCGAGTCGGTGCTTT-3’ |
| --- |
