# Supplementary material for: Chromatin accessibility is associated with CRISPR-Cas9 efficiency in the zebrafish (Danio rerio)
Source: PLoS One. 2018 Apr 23;13(4):e0196238. doi: 10.1371/journal.pone.0196238 (PMC5912780; doi:10.1371/journal.pone.0196238)
Supplement: S3 Table — (DOCX) [file pone.0196238.s003.docx]

**S3 Table. Primers used in T7 Endonuclease I assay (T7EI), Heteroduplex mobility assay (HMA) and In vitro Digestion Assay (IVDA).**

| **T7EI/HMA** | |  |  |  |
| --- | --- | --- | --- | --- |
| Gene | Accession number |  | Primer sequence (5'-3') |  |
| *ca6* | ENSDARG00000056499 | F | AGCATGCAACACCTTCGGTC |  |
|  |  | R | ATTTCAGGCATAAGTCCAGC |  |
| *ca10a* | ENSDARG00000052644 | F | CTGCAAATCATCCCTTTGTG | [29] |
|  |  | R | GTTCCTCGCATCAAAACACC |  |
| *cxcr2* | ENSDARG00000054975 | F | AGTGTTTCTCCCTCCACAGC |  |
|  |  | R | TAATGCGAAGGCTCATCCTC |  |
| *pycard* | ENSDARG00000040076 | F | GACCCAACTGTGAGGAACCATG |  |
|  |  | R | GCTTTCTTCAGACTTAAACGCCTTC |  |
| *sema4gb* | ENSDARG00000088143 | F | GGACTCACGCCTTCAGAC |  |
|  |  | R | GCCTTATATCAGCGATGTTAC |  |
| **IVDA** |  |  |  |  |
| Gene | Accession number |  | Primer sequence (5'-3') |  |
| *ca6* | ENSDARG00000056499 | F | TAGTCCACGAATGCAACAGC |  |
|  |  | R | GGCATGTCTGGCACAAATAG |  |
| *cxcr2* | ENSDARG00000054975 | F | AGTGTTTCTCCCTCCACAGC |  |
|  |  | R | GAGAAATCAGCAACTGGTTACG |  |
| *cxcr3.2* | ENSDARG00000041041 | F | GTACTCTACTCTTCCCAGGTTTACAC |  |
|  |  | R | CTGTGCGTTTTAATCTGGCA |  |
| *ifng1-2* | ENSDARG00000024211 | F | CAAAAAGATCCCCGAAGACA |  |
|  |  | R | GTGCCAGCCTCTCCTTTGTA |  |
| *pycard* | ENSDARG00000040076 | F | CAGCATTTGTGAGCAGAAGC |  |
|  |  | R | AAAGGTAGATTGGGGTGTTTG |  |
| *sema4gb* | ENSDARG00000088143 | F | ACCCCGCTGTGCTTACATAG |  |
|  |  | R | TCACTTTCATTCTGCCCAATC |  |
