# Supplementary figures and images for: Chromatin accessibility is associated with CRISPR-Cas9 efficiency in the zebrafish (Danio rerio)
Source: PLoS One. 2018 Apr 23;13(4):e0196238. doi: 10.1371/journal.pone.0196238 (PMC5912780; doi:10.1371/journal.pone.0196238)

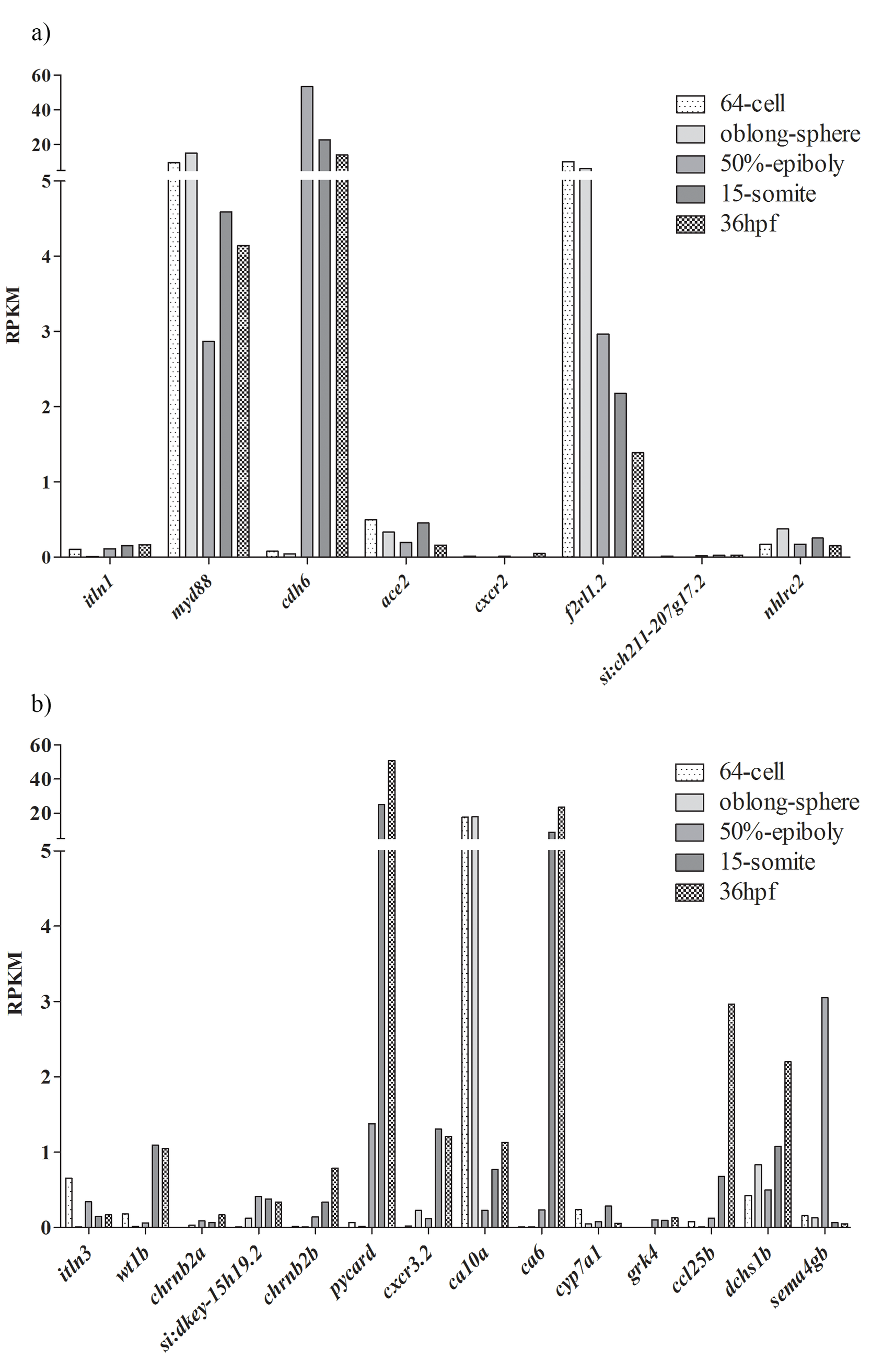

Supplement: S1 Fig — The graph a) presents the expression of the genes resistant to CRISPR-Cas9 mutagenesis, at the early stages of development. The graph b) presents the genes that were successfully mutated with CRISPR-Cas9. 2–10 sgRNAs have been used for mutagenesis. RPKM, Reads per Kilobase of transcript per Million mapped reads. All sgRNA sequences have been given in S1 Table. (TIF) [file pone.0196238.s004.tif]

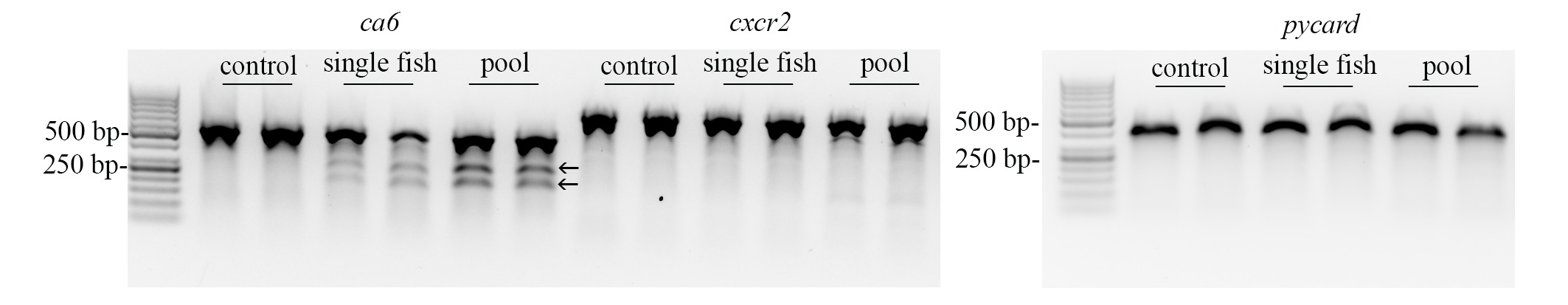

Supplement: S2 Fig — The in vivo CRISPR-Cas9 mutagenesis efficiencies for selected genes estimated with the T7EI assay for ca6, cxcr2 and pycard. 5 embryos were collected per sample at 8hpf. Black arrows indicate the mutated cleavage products for ca6. (TIF) [file pone.0196238.s005.tif]
